# Spatially fractionated radiotherapy for liver metastases: two cases of intrahepatic response dissociation

**DOI:** 10.3389/fcell.2026.1867630

**Published:** 2026-07-14

**Authors:** Yucui Zhao, Chao Li, Siyu Guo, Bicheng Zhang, Dang Wu, Qichun Wei, Ting Zhang

**Affiliations:** 1 Department of Radiation Oncology, Second Affiliated Hospital, Zhejiang University School of Medicine, Hangzhou, Zhejiang, China; 2 Cancer Institute (Key Laboratory of Cancer Prevention and Intervention, National Ministry of Education), Second Affiliated Hospital, Hangzhou, Zhejiang, China

**Keywords:** amphiregulin (AREG), hepatic microenviroment, immune remodeling, NSCLC, spatially fractionated radiotherapy (SFRT)

## Abstract

Liver is a common site of metastatic involvement from non-small cell lung cancer (NSCLC). Spatially fractionated radiotherapy (SFRT) has emerged as a promising technique for controlling bulky lesions while minimizing toxicity. We report two patients with advanced NSCLC and hepatic oligoprogression who underwent SFRT to selected liver lesions. Both achieved excellent local control within the irradiated field, yet developed explosive progression in non-irradiated hepatic regions within 3 months, while extrahepatic disease remained stable. These cases highlight a striking intrahepatic response dissociation, potentially driven by intratumoral heterogeneity, clonal selection and local hepatic immune remodeling under targeted/systemic therapy. The findings underscore the need for further investigation into SFRT’s immunobiology and optimal integration with systemic agents.

## 1 Introduction

Liver metastases are identified in ∼20% of patients with metastatic non-small cell lung cancer (NSCLC) (Zhu et al., 2024). Classified as M1c disease, these hepatic lesions are notoriously refractory to systemic therapy and confer a median overall survival (OS) of ∼4 months (Tsilimigras et al., 2021). In the CheckMate 017/057 pooled analysis, NSCLC patients with baseline liver metastases had a median OS of only 6.8 months with nivolumab versus 5.9 months with docetaxel, starkly worse than other patients without liver involvement (Borghaei et al., 2021), underscoring a profound therapeutic challenge. In patients with diffuse liver metastases, local–regional interventions such as resection or ablation are often limited by tumor burden and hepatic reserve, making liver failure a common terminal event. While stereotactic body radiation therapy (SBRT) offers a non-invasive option, its efficacy is constrained when tumors are too extensive for ablative dosing without risking radiation-induced liver disease (Mahadevan et al., 2018).

Spatially fractionated radiotherapy (SFRT) has recently re-emerged as a promising strategy for treating bulky tumors. Using a grid or lattice to create alternating high-dose “peaks” and low-to-moderate-dose “valleys”, SFRT enables dose escalation beyond conventional tissue constraints and has achieved durable local control in large thoracic and pelvic masses (Sheikh et al., 2024). From an immunological perspective, the high-dose peaks drive robust immunogenic cell death (ICD), releasing tumor-associated antigens (TAAs) and damage-associated molecular patterns (DAMPs). Concurrently, valley doses have been shown to preserve intratumoral vasculature and facilitate immune cell infiltration, enhancing dendritic cell (DC) cross-priming and CD8^+^ T-cell trafficking (Lu et al., 2023; Bekker et al., 2024). Thus, SFRT functions as an *in situ* vaccine, ideally positioned to synergize with immunotherapy (Johnsrud et al., 2020).

Preclinical studies support the potential of SFRT to induce systemic antitumor immunity and abscopal effect (Johnsrud et al., 2020), whereby non-irradiated tumors regress with systemic T-cell expansion. However, clinical abscopal responses remain rare. Emerging evidence indicates that radiotherapy can induce immunosuppressive changes, such as recruitment of myeloid-derived suppressor cells (MDSCs) and regulatory T cells (Tregs), potentially leading to “abscopal progression” (Lu et al., 2023; Wang et al., 2023). The liver, with its inherently tolerant immune microenvironment rich in Kupffer cells, PD-L1^hi^ macrophages, and Tregs, may be particularly susceptible to such remote tumor promotion (Zhu et al., 2024; Tsilimigras et al., 2021).

To date, most prospective trials combining SFRT with immunotherapy have focused primarily on immunostimulatory endpoints, with limited reporting on organ-specific abscopal progression. Here, we present two consecutive patients with NSCLC and diffuse hepatic metastases treated with lattice SFRT under the clinical trial NCT07193641. Both patients achieved prompt local tumor necrosis, yet developed explosive out-of-field hepatic progression within 3 months. These mirror-image cases highlight the clinical urgency of understanding and mitigating the potential “bad abscopal” phenomenon when SFRT is directed at hepatic metastases.

## 2 Case presentation

### 2.1 EGFR-mutant adenocarcinoma with differential hepatic response

**FIGURE 1:**
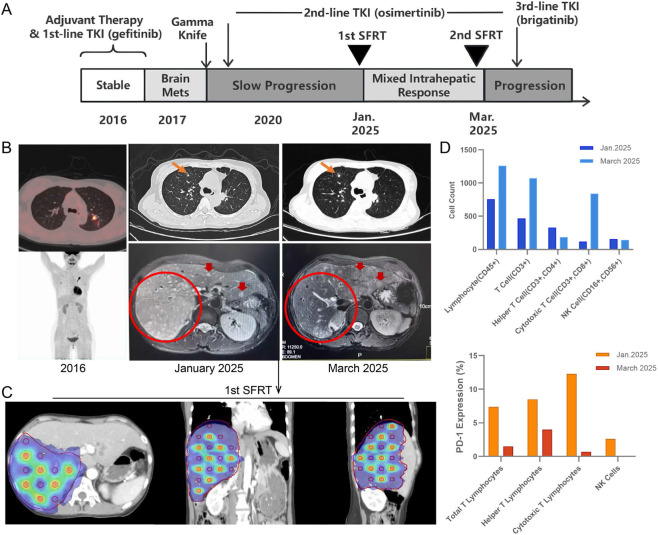
Clinical timeline, imaging, SFRT plan, and immunologic profile of Patient 1 (EGFR-mutant NSCLC). **(A)** Treatment timeline from initial diagnosis (2016) to post-SFRT follow-up (2025), showing key systemic therapies, local interventions, and disease-status landmarks. **(B)** Representative images aligned with major timeline: Baseline PET-CT (2016, left), and images before (January 2025, middle) and after (March 2025, right) the first hepatic SFRT. *Top row (Thorax):* Orange arrows showing stable pulmonary disease. *Bottom row (liver):* MRI before (middle) and after (right) the first hepatic SFRT. The irradiated dominant right-lobe metastasis (red circle) shows marked regression, while non-irradiated left-lobe lesions (red arrows) exhibit confluent progression. **(C)** Schematic of the lattice SFRT plan delivered to the right-lobe target. Orange spheres represent the high-dose PTV_lat (66.7 Gy/5 fx), and blue spheres represent the low-dose avoidance regions. Spheres (1 cm diameter) are arranged in a standardized 3-cm-spaced lattice geometry. **(D)** Peripheral-blood immunophenotyping before (January 2025) and after (March 2025) the first SFRT. Top: Absolute counts of total lymphocytes, T cells, helper T cells, cytotoxic T cells and NK cells. Bottom: PD-1 expression rates on corresponding lymphocyte subsets.

A 54-year-old woman was diagnosed with stage IIb (T2N1M0) lung adenocarcinoma in 2016, following a left upper lobectomy, adjuvant chemotherapy (carboplatin 600 mg d1 + pemetrexed 750 mg d1 q3w) and gefitinib (0.25 g once daily) for an EGFR exon 19 deletion (Figure 1A). Subsequent brain metastases in 2017 were controlled with Gamma Knife radiosurgery. Upon disease progression in 2020 with T790M mutation, therapy was switched to osimertinib (80 mg once daily). She remained on osimertinib until January 2025, when imaging revealed multiple hepatic metastases. The most prominent disease burden was in segments VI and VII of the right lobe, harboring a dominant mass (≈8.67 × 7.59 cm). Additionally, smaller metastatic lesions were noted in the left hepatic lobe (segments II, III, and IV) (Figure 1B).

On 13 January 2025, the patient underwent Lattice SFRT targeting the dominant right-lobe mass (segments VI and VII) (Figure 1C). A 6-MV single-arc volumetric modulated arc therapy (VMAT) plan was constructed with two components: a peripheral PTV receiving 20 Gy/5 fractions, and an internal lattice of twelve 1-cm spherical vertices receiving a simultaneous integrated boost of 66.7 Gy/5 fractions, arranged to achieve a peak-to-valley dose ratio >3:1 (Yan et al., 2020; Kavanaugh et al., 2022). Organ-at-risk (OAR) constraints strictly adhered to the guidelines outlined in AAPM Task Group 101 for 5-fraction SBRT (Supplementary Table S1). Treatment was well-tolerated, with grade-1 nausea and grade 1 leukopenia reported.

Post-treatment, serum carcinoembryonic antigen (CEA) level decreased from 750 ng/mL in January to 213 ng/mL in March. Peripheral blood immune profiling showed robust activation: significant increases in absolute counts of total lymphocytes, T cells, and cytotoxic T cells, alongside decreased PD-1 expression on T-cell subsets and a reversed CD4/CD8 ratio (Figure 1D). However, follow-up MRI on March 11 revealed near-complete regression of the irradiated right-lobe lesions but explosive growth of pre-existing metastases in the left lobe (segments II, III, IV), with a new dominant lesion in segment VII (Figure 1B). Liquid biopsy confirmed EGFR 19DEL, T790M, C797S and ALK G1552R mutations. A second course of SFRT was delivered to the left lobe (segments II, III, IV) on March 17, and systemic therapy was changed to brigatinib (90 mg daily) on March 20 (Figure 1A). No significant decline in liver function or constitutional symptoms was reported during second course of SFRT. Further follow-up was not available thereafter.

### 2.2 Squamous-cell carcinoma with SFRT-induced spatial disparity

**FIGURE 2:**
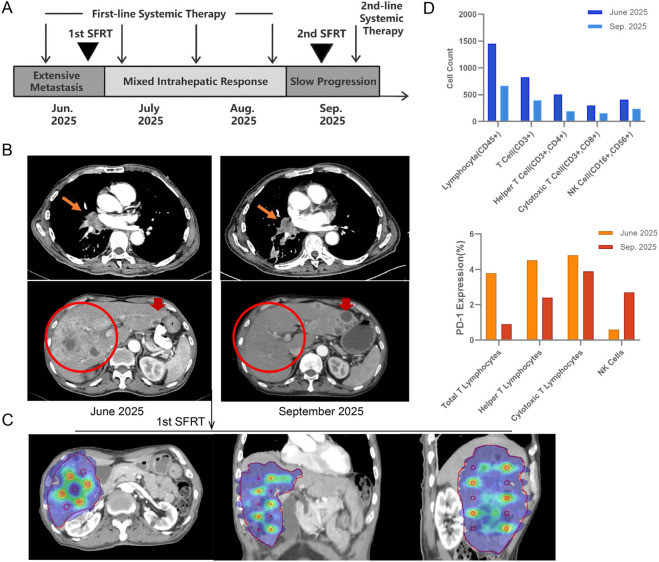
Clinical course, imaging, SFRT plan, and immune monitoring of Patient 2 (squamous-cell carcinoma). **(A)** Treatment timeline showing diagnosis, systemic therapies (chemo-immunotherapy followed by antibody-drug conjugate plus immunotherapy), local SFRT interventions, and corresponding disease-response landmarks. **(B)** Axial CT images before (June 2025, left) and after (September 2025, right) the first hepatic SFRT. *Top row (chest):* stable hilar lymphadenopathy (orange arrows). *Bottom row (liver)*: irradiated right-lobe lesions (red circles) show pronounced regression, while untreated left-lobe metastases (red arrows) demonstrate progressive growth. **(C)** Schematic of the lattice SFRT plan delivered to the dominant right-lobe metastases. The plan comprised an internal high-dose lattice (orange spheres, PTV_lat, 66.7 Gy/5 fx) embedded within a peripheral target volume (blue spheres, PTV_20, 20 Gy/5 fx), conforming to the same standardized geometry as in Figure 1C. **(D)** Changes in absolute counts of key lymphocyte subsets (top) and PD-1 expression rates on the corresponding lymphocyte subsets (bottom) between June (pre-1^st^ SFRT) and September (post-1^st^ SFRT) 2025.

A 70-year-old man presented in June 2025 with squamous-cell carcinoma of the right middle lobe of the lung and extensive metastatic disease involving multiple bony sites and diffuse liver metastases, with the largest burden in segments V, VI, and VIII (Figures 2A,B). First-line systemic therapy with chemo-immunotherapy (Endostar 75 mg d1-3, Albumin-paclitaxel 400 mg d1, Carboplatin 400 mg d1, Sintilimab 200 mg d1 q3w) was administered for four cycles from 10 June to 25 August 2025. On 30 June 2025, SFRT was initiated targeting the two largest lesions in the right liver (segments V, VI, and VIII) (Figure 2C). A 6-MV VMAT plan was generated with a standardized lattice approach. The plan similarly consisted of a peripheral PTV_20 (20 Gy/5 fx) and an internal lattice PTV_lat (eight 1-cm spheres, 66.7 Gy/5 fx). Treatment was well-tolerated, with no acute toxicities exceeding Grade 1.

Follow-up immune profiling in September indicated progressive exhaustion, with comprehensive declines in lymphocyte, T cell, helper T cell, and cytotoxic T cell counts. PD-1 expression rates across T-cell subsets decreased in the context of global lymphopenia, while PD-1 expression on NK cells increased paradoxically (Figure 2D). Surveillance CT on 14 September showed marked regression of the irradiated right-liver lesions. However, progressive disease was noted in the untreated left lobe (segments II, III, IV) and superior right lobe (segments VII, VIII); all extrahepatic disease sites remained stable (Figure 2B). A second SFRT course was initiated for the progressive lesions but was discontinued after 3 fractions due to abdominal bloating. Systemic therapy was subsequently switched to sacituzumab govitecan (200 mg) plus ivonescimab (1,200 mg), initiated on 22 September. One week after starting the new regimen, the patient again experienced abdominal bloating.

## 3 Discussion

SFRT achieves potent local control of bulky tumors (Zhang and Wu, 2024; Prezado et al., 2024), as evidenced by the marked radiographic regression in our patients, supporting its therapeutic rationale in oligoprogressive disease—a central premise of our trial (NCT07193641). Aggressive local treatment to the liver was pursued with the intent to consolidate local control, potentially reverse emerging systemic resistance, and prolong the benefit of existing systemic therapy, consistent with our trial paradigm. However, both patients experienced rapid and explosive progression in unirradiated hepatic segments within 90 days. Notably, extrahepatic disease sites remained relatively stable during this period, suggesting a liver-specific progression pattern. Retrospective review of liver diffusion-weighted imaging revealed subtle baseline diffusion restriction in regions that later progressed (Supplementary Figure S1), indicating the presence of pre-existing micrometastases. Moreover, both patients harbored multiple resistance mutations (e.g., EGFR T790M/C797S, ALK G1552R), underscoring the dominant role of tumor clonal heterogeneity and selective outgrowth of drug-resistant clones. While these factors likely represent the primary drivers, a potential modulating influence of the irradiated hepatic microenvironment cannot be excluded and merits consideration.

The observed discordant hepatic progression, while multifactorial, prompts the hypothesis that high-dose SFRT may have contributed to a local immune microenvironmental shift—a phenomenon reminiscent of the “badscopal” effect described in rigorous preclinical models (Piffko et al., 2025). While SFRT is designed to create steep “peak-and-valley” dose gradients that enhance ICD and antigen presentation (Jagodinsky et al., 2024), the high-dose component may simultaneously initiate that, in preclinical systems, can promote metastatic outgrowth (Piffko et al., 2025). Recent work identifies a “danger zone” of high single-fraction-equivalent doses (e.g., 20 Gy) that robustly induce AREG secretion from irradiated tumor cells (Piffko et al., 2025). AREG can acts on EGFR^+^ liver mononuclear phagocytes (MNPs), including Kupffer cells (Mulder et al., 2021), potentially polarizing them toward an immunosuppressive phenotype. Based on our supplemented comprehensive dosimetric analysis (Supplementary Table S2), The lattice dose in our SFRT plan (66.7 Gy/5 fx) corresponds to an EQD_2_ of 30–34 Gy in a single fraction (α/β = 10 Gy), which lies within the reported “danger zone”. Thus, the rapid out-of-field progression observed in our cases may, in part, reflect an AREG-influenced process that subverts a potential abscopal response, although this connection remains speculative without direct tissue evidence.

Our attempt to explore this mechanism revealed a nuance. To investigate the role of systemic AREG, we measured peripheral blood AREG levels. In Case1, the level was only 10.66 pg/mL at the time of hepatic progression (3 months post-first SFRT). Furthermore, an expanded ELISA cohort analysis (including healthy controls, tumor baseline patients, and post-SFRT patients) showed no significant differences in peripheral AREG concentrations among groups (Supplementary Figure S2). This negative finding, coupled with the observed systemic T-cell expansion and PD-1 downregulation in Patient 1 alongside stable extrahepatic disease, suggests that sustained high-level systemic AREG may not be the sole or primary driver. These divergent patterns of immune profiling in Case 2 illustrate the inherent limitation of PD-1 expression as a solitary marker—it cannot reliably distinguish activation from exhaustion without additional phenotypic or functional characterization. Moreover, the interpretation in Case 2 is confounded by concurrent chemoimmunotherapy and subsequent systemic treatment changes. Therefore, we suspect that any AREG-mediated immunomodulation is anatomically confined to the liver. This hypothesis does not require elevated systemic AREG; it only requires that high-dose irradiation triggers local AREG release within the tumor microenvironment, which then acts on adjacent EGFR-expressing liver mononuclear phagocytes, including Kupffer cells, in a paracrine manner, as demonstrated in preclinical models (Ciner et al., 2021). The liver’s inherent immune-tolerant nature—shaped by Kupffer cells and DCs that constitutively promote Treg expansion and express high levels of PD-L1, TGF-β, and IL-10 (Zhu et al., 2024; Ciner et al., 2021)—provides fertile ground for such localized suppression. In this model, SFRT-induced AREG may act as a catalyst that acutely exacerbates this tolerogenic “soil”, locally overriding systemic anti-tumor immune signals and confining any potential abscopal activity to the irradiated lobe (Yu et al., 2021). We acknowledge that this localized hypothesis remains entirely inferential in the absence of direct intrahepatic protein measurements or tissue-level immune profiling, and it should be regarded strictly as a hypothesis-generating construct to be tested in future studies with invasive hepatic sampling.

These observations warrant prospective studies to better understand the biological mechanisms underlying this phenomenon. Future strategies should consider real-time monitoring of the hepatic immune landscape (e.g., via macrophage-targeted PET-CT (Gondry et al., 2023)) or functional MRI—to capture the dynamics of myeloid dysfunction before anatomical progression occurs. Furthermore, combining SFRT with agents that simultaneously target local immunosuppression (e.g., AREG/EGFR blockade (Piffko et al., 2025)) and radiation-induced tumor plasticity (Wang et al., 2025) represents a rational, testable approach. An additional dosimetric question raised by our cases is whether de-escalating the high-dose spheres to remain below the AREG-induction “danger zone,” while concurrently delivering a very low-dose prophylactic whole-liver irradiation, could improve control of out-of-field hepatic micrometastases without triggering a local tolerogenic surge. This remains a hypothesis for future trials to explore. Such an integrated strategy, guided by local microenvironmental imaging and grounded in the physical precision of SFRT, may help transform discordant hepatic progression into a meaningful systemic response.

## Data Availability

The raw data supporting the conclusions of this article will be made available by the authors, without undue reservation.
