# Supplementary material for: Spatially fractionated radiotherapy for liver metastases: two cases of intrahepatic response dissociation
Source: Front Cell Dev Biol. 2026 Jul 14;14:1867630. doi: 10.3389/fcell.2026.1867630 (PMC13408373; doi:10.3389/fcell.2026.1867630)
Supplement: Supplementary file 1 [file DataSheet1.docx]

**Supplementary Appendix**

**Supplementary Table 1. Dose-volume constraints for critical organs at risk applied over the entire 5-fraction SFRT course in Case 1 and 2.**

| **OAR metrics** | **Actual value of Case 1** | **Actual value of Case 2** | **Dose constraints** |
| --- | --- | --- | --- |
| Spinal cord | Dmax = 15.99 Gy | Dmax = 10.87 Gy | Dmax ≤ 30 Gy |
| Esophagus | Dmax =13.38 Gy | Dmax =11.74 Gy | Dmax ≤ 35 Gy |
| Stomach | Dmax = 11.23 Gy | Dmax = 4.22 Gy | Dmax ≤ 32 Gy |
| Duodenum | Dmax = 18.43 Gy | Dmax = 0.78 Gy | Dmax ≤ 32 Gy |
| Small intestine | Dmax = 18.94 Gy | Dmax = 0.35 Gy | Dmax≤ 35 Gy |
| Colon | Dmax = 0.99 Gy | Dmax = 0.99 Gy | Dmax ≤ 38 Gy |
| Liver  (uninvolved) | Volume=1029.9 cc  V≤21 Gy=978.4 cc | Volume =1068.5 cc  V≤21 Gy= 751.8 cc | V_≤21 Gy_> 700 cc |
| Left kidney | Total volume =162.9 cc  V≤ 17.5 Gy =0 cc | Total volume = 229.5 cc  V≤ 17.5 Gy =0 cc | V_≤17.5 Gy_> 200 cc |
| Right kidney | Total volume =156 cc  V≤ 17.5 Gy=14.77 CC | Total volume = 204.9 cc  V≤ 17.5 Gy= 0 CC | V_≤17.5 Gy_> 200 cc |

**Supplementary Table 2. Target coverage and gradient metrics for Case 1 and 2.**

| **Metric Category** | **Metric Name** | **Unit** | **Case1 Measured Value** | **Case2 Measured Value** | **Remarks** |
| --- | --- | --- | --- | --- | --- |
| Liver DVH Metrics | Liver V20Gy | % | 1.4 | 20 | PTV_2000 prescription coverage requirement |
|  | Liver Dmean | Gy | 8.1 | 12.3 | Mean dose constraint for liver |
|  | Liver Dmax | Gy | 27.1 | 68.6 | Maximum point dose constraint for liver |
| Cumulative Liver Dose | Cumulative Liver V21Gy | cc | 14.4 | 213.6 | Full-course cumulative dose constraint, 5 fractions |
|  | Cumulative Liver D90 | % | 2 | 2.5 | Dose covering 90% of liver volume |
|  | Cumulative Liver D50 | % | 6.5 | 12 | Dose covering 50% of liver volume |
| Valley-Dose Characterization | PTV_Avoid Mean Dose | Gy | 22.4 | 22 | Valley dose prescription target |
|  | PTV_Avoid V18Gy | % | 100 | 100 | Valley dose minimum coverage requirement |
| Dose Exposure of Progressing Lesions | Peak-Valley Dose Ratio (DR) |  | 3.3 | 3.2 | Core lattice metric: PTV_6670 Dmean / PTV_Avoid Dmean |
|  | Progressing Lesion Dmean | Gy | 2.9 | 4.5 | Mean dose delivered to the progressing lesion |
|  | Progressing Lesion Dmax | Gy | 3.5 | 6.1 | Maximum point dose in the progressing lesion |
| Spatial Mapping of Progression Relative to Dose Gradients | Progression Zone Classification |  | low-dose valley | low-dose valley | High-dose peak / 1.5cm gradient ring / low-dose valley |
|  | Distance from Progression to Nearest High-Dose Sphere | cm | 11.1 | 11.5 | Spatial distance between lesion and lattice high-dose region |
|  | Dose Gradient at Progression Site | Gy/cm | 0.45 | 0.47 | Dose falloff rate at the lesion location |

**Supplementary Figure 1.** **Pre- (Jan 2025, left) and post- SFRT (Mar 2025, right) diffusion-weighted MR images of Case 1:** irradiated right-lobe lesions (red circles) achieve prominent regression, and untreated left-lobe metastases (red arrows) progress in size.


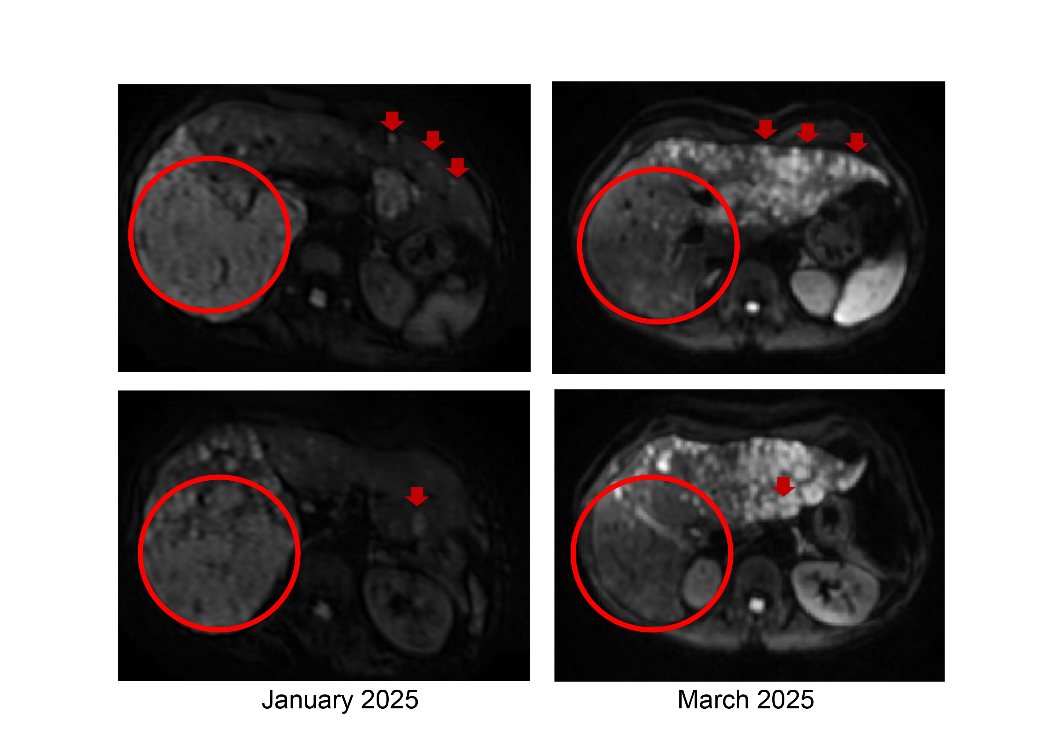


**Supplementary Figure 2. Serum AREG concentrations across cohorts.** Levels were measured by ELISA in healthy controls (n=5), treatment-naïve tumor patients at baseline (n=12), and patients within 1–4 months after SFRT (n=5). Differences were assessed by one-way ANOVA. (ELISA kit: Boster Biological Technology, EK0304).
